# Use of Polyhedral Oligomeric Silsesquioxane (POSS) in Drug Delivery, Photodynamic Therapy and Bioimaging

**DOI:** 10.3390/molecules26216453

**Published:** 2021-10-26

**Authors:** Paula Loman-Cortes, Tamanna Binte Huq, Juan L. Vivero-Escoto

**Affiliations:** 1 Department of Chemistry, The University of North Carolina at Charlotte, Charlotte, NC 28223, USA; plomanco@uncc.edu (P.L.-C.); thuq@uncc.edu (T.B.H.); 2 Nanoscale Science Program, The University of North Carolina at Charlotte, Charlotte, NC 28223, USA; 3 The Center for Biomedical Engineering and Science, The University of North Carolina at Charlotte, Charlotte, NC 28223, USA

**Keywords:** polyhedral oligomeric silsesquioxane (POSS), drug delivery systems (DDS), photodynamic therapy (PDT), biomedical applications, imaging

## Abstract

Polyhedral oligomeric silsesquioxanes (POSS) have attracted considerable attention in the design of novel organic-inorganic hybrid materials with high performance capabilities. Features such as their well-defined nanoscale structure, chemical tunability, and biocompatibility make POSS an ideal building block to fabricate hybrid materials for biomedical applications. This review highlights recent advances in the application of POSS-based hybrid materials, with particular emphasis on drug delivery, photodynamic therapy and bioimaging. The design and synthesis of POSS-based materials is described, along with the current methods for controlling their chemical functionalization for biomedical applications. We summarize the advantages of using POSS for several drug delivery applications. We also describe the current progress on using POSS-based materials to improve photodynamic therapies. The use of POSS for delivery of contrast agents or as a passivating agent for nanoprobes is also summarized. We envision that POSS-based hybrid materials have great potential for a variety of biomedical applications including drug delivery, photodynamic therapy and bioimaging.

## 1. Introduction

Hybrid materials have attracted tremendous attention recently due to their potential applications in several fields such as energy, biomedicine, materials, catalysis, and others [1,2,3,4]. Hybrid materials combine the advantages of both organic and inorganic components to develop unique high-performance materials [5,6]. 

**Scheme 1:**
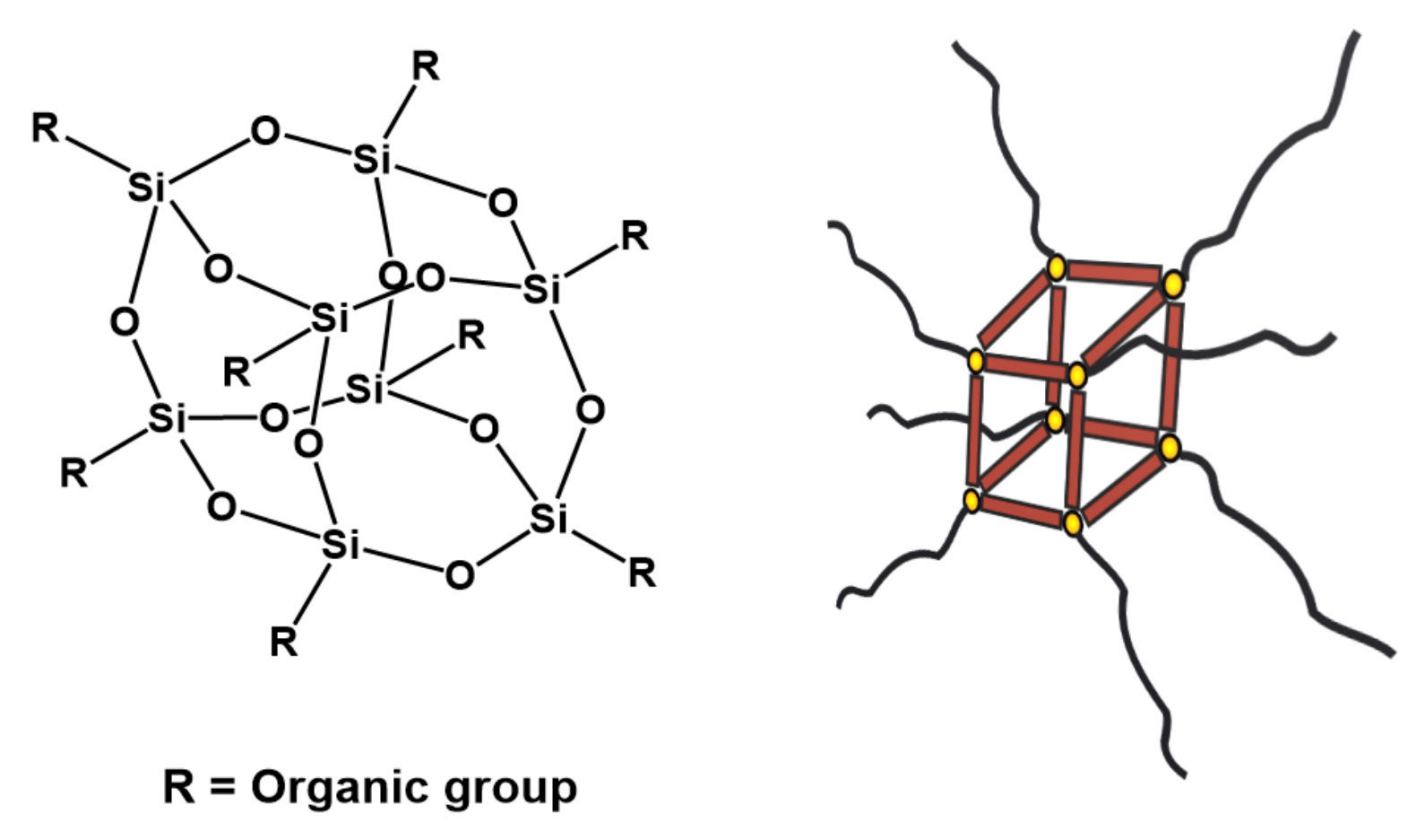
Schematic representation of POSS as a cube where the Si atoms are localized in the corner. R groups are substituents to the POSS that can tune its physicochemical properties or be used for further chemical modification.

A polyhedral oligomeric silsesquioxane (POSS) is a class of hybrid materials with unique 3D configuration, definite framework, and customizable physicochemical properties. POSS may be considered as the smallest possible form of hybrid silica-based materials with sizes of 1–3 nm [7,8]. The basic chemical structure of POSS has the composition of (RSiO_1.5_)_n_ (*n* = 6, 8, 10, 12, …), where R represents either a hydrogen atom or organic groups. The most common form of POSS is *n* = 8, also known as a POSS cage. In this review, we will use the words POSS cage and POSS interchangeably, unless otherwise specified. POSS molecules consist of a core cubic cage of eight silicon corner atoms and twelve oxygen edge atoms (Si-O-Si), where each of the silicon atoms may carry an organic group surrounding the periphery of the cage. These groups can be further modified to afford hundreds of possible compounds. POSS combine many of the benefits of silica (thermal, chemical and radiation stability, and optical transparency) with those of siloxane polymers (solubility, low toxicity), as well as the potential to further modify their structure by functionalizing R to achieve the desired properties (Scheme 1). All these features make POSS a unique and versatile platform to produce high-performance multi-functional hybrid materials [9].

In the past decades, POSS have been used as building blocks, crosslinkers, and nanofillers to afford hybrid materials with improved stability, mechanical properties and chemical resistance [10]. POSS have great potential in various fields such as catalysis, sensing, photoelectronic, energy, and biomedicine [3,11,12,13,14,15]. In this review, we will emphasize the most recent advances on the use of POSS in biomedicine, with particular focus on drug delivery, photodynamic therapy and bioimaging.

## 2. Engineering POSS 

POSS typically have rigid 3D nanosized structures with a diameter of approximately 1–3 nm, which provides several advantages to POSS monomers in building sophisticated hybrid materials [5]. In fact, the organic groups on POSS molecules can be precisely defined, which helps to easily verify the quantity and spatial position of functional groups. In addition, the rigid silica core endows POSS molecules with favorable mechanical/thermal/chemical stability; while the features of the organic groups on the periphery have a significant impact on the molecule packing and dispersion of POSS [9]. 

The functionalization of POSS molecules can render endless possibilities to engineering materials with a desired performance. A wide variety of functional groups, such as olefins, acrylates, phenols, fluoroalkyls, halides, amines, sulfhydryls, azides, nitriles, phosphines, carbazoles, imidazolium salts, azobenzenes, cinnamates, etc., can be used for this purpose [4,7]. Some examples of current applications of these functionalized POSS are: phenyl functionalized POSS are promising as gas separation membranes or as surface coatings for electronic or optical devices owing to their excellent thermal stability and electrical insulation [16,17]; vinyl-functionalized POSS can be grafted to or copolymerized with polymers because of the unsaturated groups on the surface [18]; amino-functionalized POSS hybrid composites show high reactivity and oxidation resistance [19,20]; and mercapto-functionalized POSS can adsorb proteins or metal ions [21,22]. The functionalization of POSS is of great significance for the subsequent design and synthesis of intermediates and materials. 

Depending on the molecular design requirements and reactivity of corner substituents, POSS could incorporate into a polymer matrix as grafted chains or act as cross-linking sites. The recent development of controlled/living radical polymerization techniques, such as atom transfer radical polymerization (ATRP), reversible addition-fragmentation chain transfer polymerization (RAFT), ring-opening metathesis polymerization (ROMP), and anionic polymerization allow for a better control of the polymerization process. The accessibility to different functional groups, in combination with click chemistry, has allowed researchers to prepare reactive POSS more efficiently to develop high-performance hybrid materials. These polymerization strategies have been used to fabricate polymer POSS with different architectures, including tadpole-, dumbbell-, multi-telechelic, star-like, and dendritic [3,4,13]. 

**Scheme 2:**
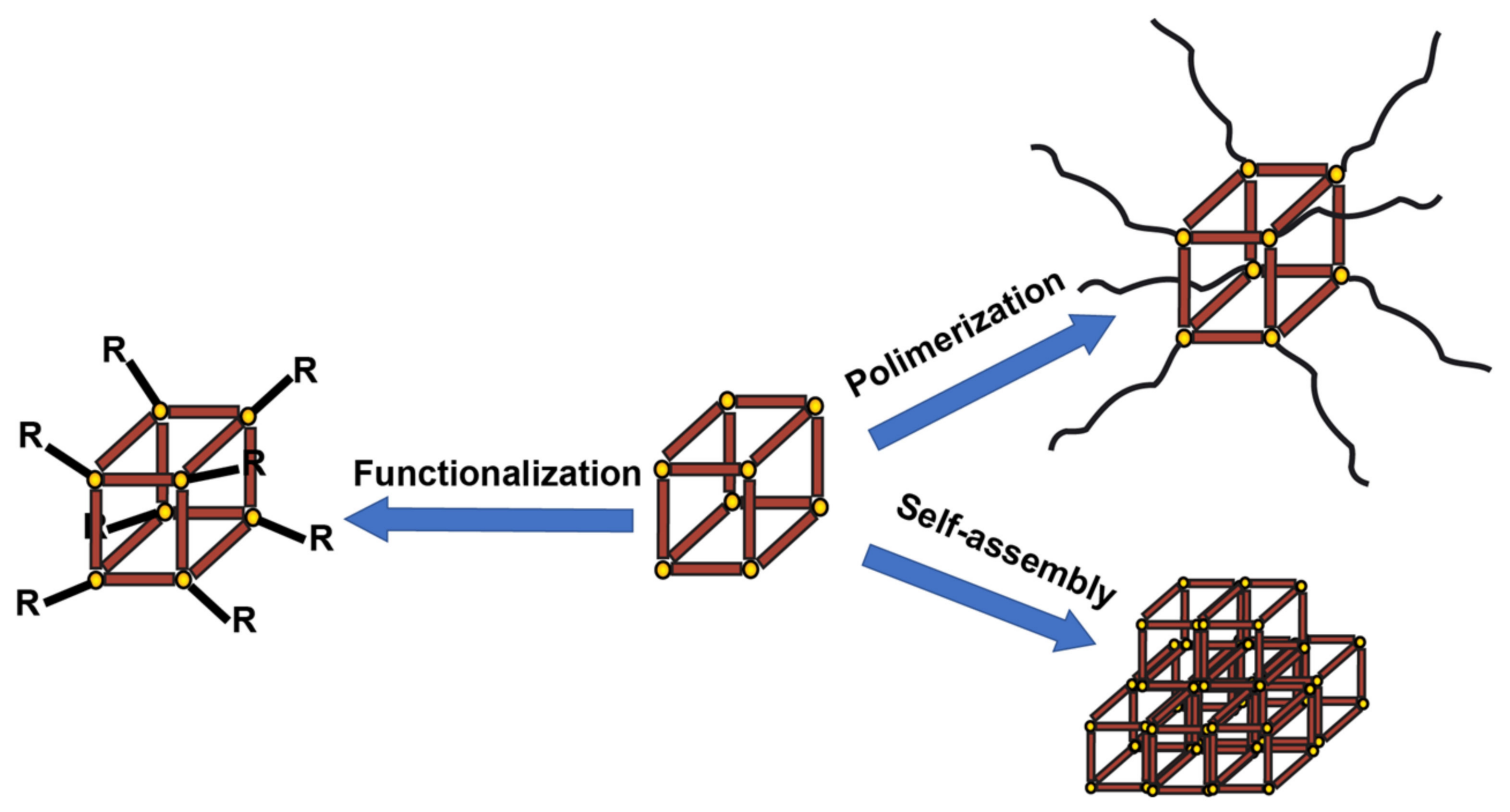
The physicochemical properties of POSS can be engineered through functionalization, polymerization and/or self-assembly.

In recent years, the use of building blocks that can be precisely designed to fabricate hybrid materials through self-assembly has been a burgeoning field of research. The hydrophobicity and low surface energy of POSS provides a strong tendency to aggregate, which contributes to the controlled self-assembly and confined motion of polymer chains. In turn, these properties lead to the desired topologies and physicochemical properties [23,24]. In addition, POSS can also be employed as building blocks for the precise synthesis of giant molecules through co-functionalization with surfactants or amphiphiles that significantly improve the compatibility of POSS with polymer moieties [25,26]. Self-assembly of such giant molecules is typically driven by a combination of attractive and repulsive forces between POSS and polymer segments. Scheme 2 summarizes some of the main strategies for engineering POSS.

## 3. Synthesis and Functionalization of POSS 

### 3.1. Sol-Gel Synthetic Protocols for POSS

**Scheme 3:**
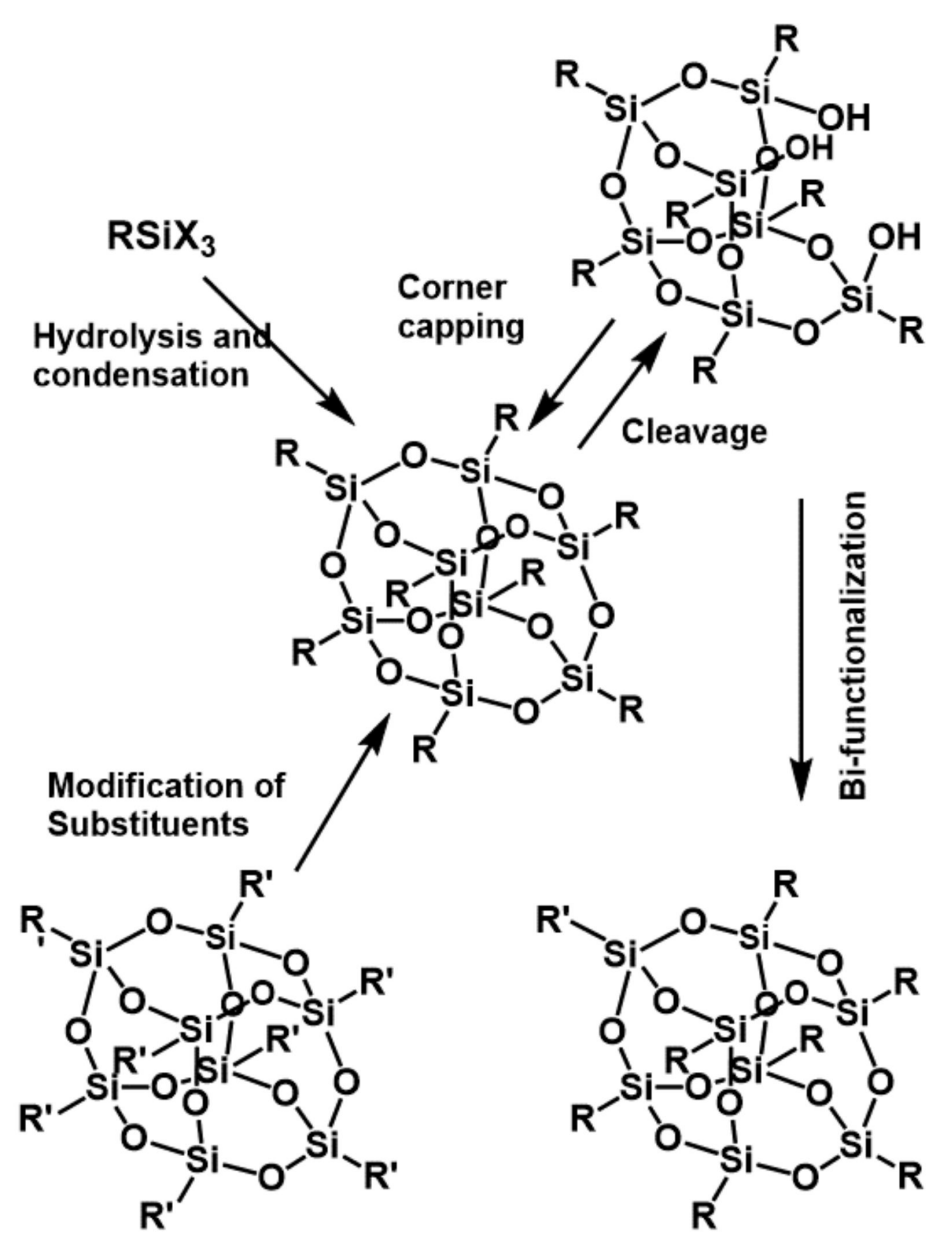
Synthetic strategies for the fabrication of POSS derivatives.

The most basic synthesis to afford completely or partially condensed POSS is the sol-gel method in the presence of RSiCl_3_ or RSi(OR’)_3_ precursors (Scheme 3). This synthetic approach can be described as a multistep hydrolysis/condensation pathway. A wide variety of monofunctionalized POSS molecules have been obtained using this approach with functional groups such as alkyl, vinyl, aromatics, amino, thiol, etc. [7]. Using this approach, our group synthesized octa(aminopropyl)-POSS using APTES as a silica precursor. The octa(aminopropyl)-POSS was reacted with carboxy(phenyl)-triphenyl porphyrin via an amidation reaction to afford a porphyrin-POSS molecule [27]. Also, Hörner and co-workers used octa(aminopropyl)-POSS as a starting material to be modified with fluorescent tetramethyl-rhodamide. Subsequently, the authors changed the ammonium groups with other functionalities to test the cellular uptake of all POSS derivatives [28].

### 3.2. Corner Capping of Partially Condensed POSS

Most POSS-bearing compounds used for biomedical applications are made by modifying an existing cage or partially condensed POSS through the corner capping reaction (Scheme 3). The POSS structures are synthesized by hydrolytic condensation of RSiCl_3_ or RSi(OR’)_3_ precursors, many of which are commercially available. This reaction generally affords good yields and POSS molecules with a wide variety of functional groups such as amino, thiol, vinyl, halogen, etc. For instance, our group used this strategy to afford aminopropyl-hepta(isobutyl)- or aminopropyl-hepta(phenyl)-POSS from the corresponding trisilanol POSS precursors. These molecules were further modified to finally obtain porphyrin-POSS molecules [27]. Using the corner capping strategy, Ervithayasuporn and co-workers reacted 3-chloropropyltrhichlorosilane with hepta(isobutyl)-trisilanol POSS to obtain a cage with only one chloropropyl functionality, that was later subjected to an azidation with excess NaN_3_ in DMF-THF (93% yield). The azido group can later be used for “click chemistry” [29].

### 3.3. Cleavage of Completely Condensed POSS

POSS cages can be opened in either basic or acidic conditions. The optimization of the consecutive cleavage and corner-capping steps opens the possibility of the insertion of two distinct functionalities to afford bifunctional POSS (Scheme 3). Following this procedure, POSS containing both amino and carboxylic acid groups were fabricated for further functionalization with luminescent moieties with potential biomedical applications [30]. Olivero and co-workers synthesized a POSS molecule with six isobutyl, one FITC derivative, and one carboxylic functionalized substituents. They used as a starting material aminopropyl hepta(isobutyl)-POSS to open a corner under basic conditions to obtain trisilanol aminopropyl hexa(isobutyl)-POSS. Then they reacted, through a corner capping reaction, a fluorescein silane derivative (FITC-APTES) with trisilanol aminopropyl hexa(isobutyl)-POSS to afford the bifunctional amino-, fluorescein-POSS molecule. The aminopropyl group was subsequently modified by the reaction with succinic anhydride to add a carboxylic acid termination on this substituent. The resulting molecule was able to enter the HeLa cells cytosol without cytotoxicity [31]. 

### 3.4. Functionalization of POSS

Several reviews and books have already been published on the synthesis, characterization and functionalization of POSS molecules. Therefore, a complete overview of this topic is out of the scope of this review [3,4,5,7,9,12]. Nevertheless, we will focus on several practical approaches recently used for POSS functionalization with biomedical applications.

The functionalization of peripheral groups of POSS is facilitated by the use of the so-called click chemistry [30,32]. Click reactions include several kinds of selective and orthogonal chemical ligations with high efficiency under mild reaction conditions. Typical click reactions include Cu(I)-catalyzed [3 + 2] azide–alkyne cycloaddition (CuAAC), strain-promoted azide–alkyne cycloaddition (SPAAC), Diels–Alder cycloaddition, thiol-ene addition/thiol reaction (TEC), and oxime ligation. Some clickable groups are alkynes, cyclooctynes, alkenes, azides, thiols, amines, etc. Fabritz and co-workers used click chemistry to attach a single fluorescein group to POSS octa azide using a copper-catalyzed azide-alkyne cycloaddition (CuAAC), leaving an amide linker [33].

Another widespread modification strategy to produce POSS molecules with biomedical applications is the thiol–ene click chemistry. This approach relies on the availability of vinyl-containing POSS such as octa(vinyl) POSS or mono-substituted vinyl POSS. The thiol–ene reaction shows several advantages: it is quick, tolerant to water and oxygen present in the environment, requires mild conditions, uses low-cost catalysts, and leads to high yields [30,32]. Several examples have been published in the literature on using thiol–ene chemistry to functionalize POSS molecules for potential biomedical use. Fan and co-workers obtained hepta(vinyl)-mono(hydroxyl)-POSS by reacting octa(vinyl)-POSS with trifluoromethanesulfonic acid. The obtained product was reacted with 1-adamantanecarbonyl chloride. The remaining seven vinyl arms were reacted using thiol–ene chemistry with a thiol-terminated zwitterionic chain under UV irradiation. The adamantane arm was used to complex with a star-shaped host molecule based on cyclodextrin and polylactic acid. The whole complex was self-assembled and loaded with doxorubicin (DOX), an anticancer drug [34]. In another example of the use of thiol–ene chemistry, Han and co-workers obtained water-soluble ionic star POSS from octa(vinyl)-POSS through sequential reactions using thiol–ene addition, quaternization and the introduction of a terminal alkyne functionality followed by copper-catalyzed azide-alkyne cycloaddition (CuAAC) to insert long hydrophobic chains. The resulting amphiphile was used to encapsulate dyes very efficiently for in vitro delivery [35]. Gao and others also used thiol–ene chemistry to conjugate the cell penetrating peptide TAT with octa-diallyl POSS to obtain a star-shaped molecule that could form small micelles in aqueous solutions [23,36]. 

### 3.5. POSS as Monomers for Fabricating Hybrid Materials

POSS molecules impart functional properties such as structural stability, processability, optical properties, low toxicity, and biocompatibility to polymer-POSS hybrids. Factors such as POSS content, POSS functionalization, polymer-POSS interaction, and mode of cross-linking influence the final hybrid structure [24,37]. Most of the strategies described above have been used for the functionalization of POSS compounds to afford POSS monomers. For example, a capping reaction has been utilized to introduce monomers like methylacrylate to POSS molecules using trichlorosilane as a capping agent. Similarly, other functional groups such as styryl or vinyl monomers may be introduced on the surface of POSS via corner-capping reactions using coupling agents. Several well-known polymerization techniques are used to fabricate polymer-POSS hybrids, such as ATRP, ROMP, RAFT, and click chemistry [24]. These techniques have shown their value, particularly in the formation of self-assembled polymers and block copolymer hybrids with functional POSS molecules for biomedical applications. For instance, Ma and co-workers created a POSS-Br initiator by reacting 2-bromoisobutyryl bromide with aminopropyl heptakis(isobutyl) POSS, which was then reacted with 2-(dimethylamino)ethyl methacrylate via ATRP to yield a POSS-capped poly[2-(dimethylamino)ethyl methacrylate] (POSS–PDMAEMA). The polymer has an interesting pH-dependent self-assembly behavior [38]. Wu and co-workers used the same initiator to attach PDMAEMA on POSS, followed by a layer of polymethyl methacrylate (PMMA), creating a POSS-PDMEAMA-PMMA polymer that, after self-assembly in water, could encapsulate the fluorophore tetraphenylethene and facilitate its cellular uptake [25]. Yang and collaborators made a star POSS with poly(2-dimethylamino)ethyl methacrylate (PDMA) arms linked to the core by disulfide bonds which are cleavable under physiological conditions. Starting from octa(vinyl)-POSS, they reacted with cysteamine hydrochloride and 4-(dimethylamino)-pyridine under UV irradiation. The resulting terminal amine group was reacted with succinic anhydride to attach a terminal carboxylic acid functionality. Then, the initiator for the ATRP reaction and the disulfide bond were introduced by an esterification reaction with 1-(3-dimethylaminopropyl)-3-ethylcarbodiimide hydrochloride and 2-bromo-2-methyl-propionic acid 2-(2-hydroxy-ethyldisulfanyl)-ethyl ester. Finally, a typical ATRP polymerization method was carried out to grow the polyacrylate arms with the addition of DMAEMA and HMTETA and copper(I) bromide as a catalyst. This type of star POSS polymer was shown to enhance the delivery of genes [21]. Using a similar approach, Wang and co-workers reacted octa(3-ammoniumpropyl) POSS with 2-bromoisobutyryl bromide and used it to initiate ATRP polymerization with DMAEMA and poly(ethylene glycol)monomethacrylate (PEGMA) under CuBr. This synthetic step was followed by a second ATRP using PEGMA. The hydroxy groups of PEGMA were functionalized with double bonds by reacting with acryloyl chloride and then peptide chains were coupled using the thiol–ene click reaction. The system was used for DNA transfection of endothelial cells [39]. Ray et al. [35] used commercially available mercaptopropyl hepta(isobutyl) POSS to react with acetylene-functionalized poly(benzyl-l-glutamate) via thiol–ene chemistry. The resulting pH-responsive A2B star polymer can self-assemble in an aqueous solution forming vesicles [40].

This section describes just some examples of the different synthetic strategies to afford POSS with particular chemical/physical properties for biomedical applications. In Table 1, below, a comprehensive table is provided to illustrate the wide variety of approaches that have been utilized to functionalize POSS.

## 4. Applications of POSS for Drug Delivery

The development of efficient drug delivery platforms is critical to implementing current advances in diverse areas of medicine. Unfortunately, many drugs with a good therapeutic effect are disregarded because of the lack of bioavailability in clinical trials. A drug delivery system (DDS) can be defined as a formulation or material that enables the administration of a therapeutic drug into the body, improving its bioavailability, efficacy and safety. In the past decades, nanoparticles have been explored as an excellent option to develop highly efficient DDS. In particular, silica-based hybrid nanomaterials such as mesoporous silica nanoparticles (MSNs) [84,85,86], polysilsesquioxane nanoparticles (PSilQ NPs) [87,88,89,90,91] and POSS have been explored for this purpose [13,23,26]. These silica-based platforms have been utilized to deliver chemotherapeutic drugs, proteins, genes and photosensitizers. Of these silica-based platforms, POSS offer several unique advantages, such as easy functionalization and biocompatibility, which allow the development of DDS with improved therapeutic efficacy and reduced side-effects.

### 4.1. Chemotherapy

POSS can be used to efficiently transport anticancer drugs through cell membranes, either as individual entities or as self-assembled nanomaterials. In addition, POSS can be further functionalized with targeting agents and/or polymers to enhance its targeting ability toward cancer cells and its colloidal stability in physiological conditions. Below we describe some examples that illustrate the use of POSS for chemotherapeutic applications.

**Figure 1:**
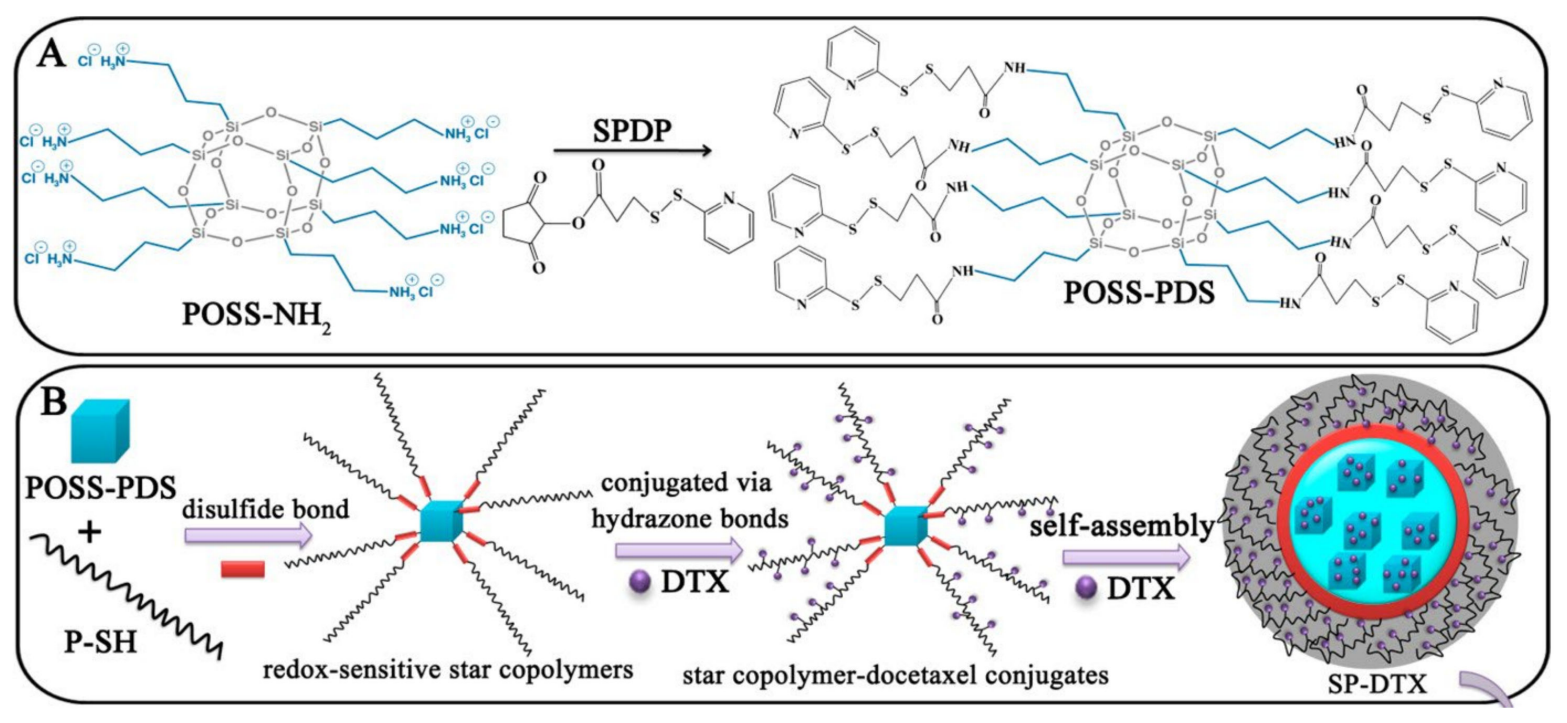
(**A**) Synthesis procedure of pyridyldisulfanyl-functionalized POSS (POSS−PDS). (**B**) Fabrication of SP-DTX nanoparticles which self-assembled from amphiphilic star-shaped POSS-based conjugates. Reprinted with permission from ref. [92]. Copyright 2016 American Chemical Society.

**Figure 2:**
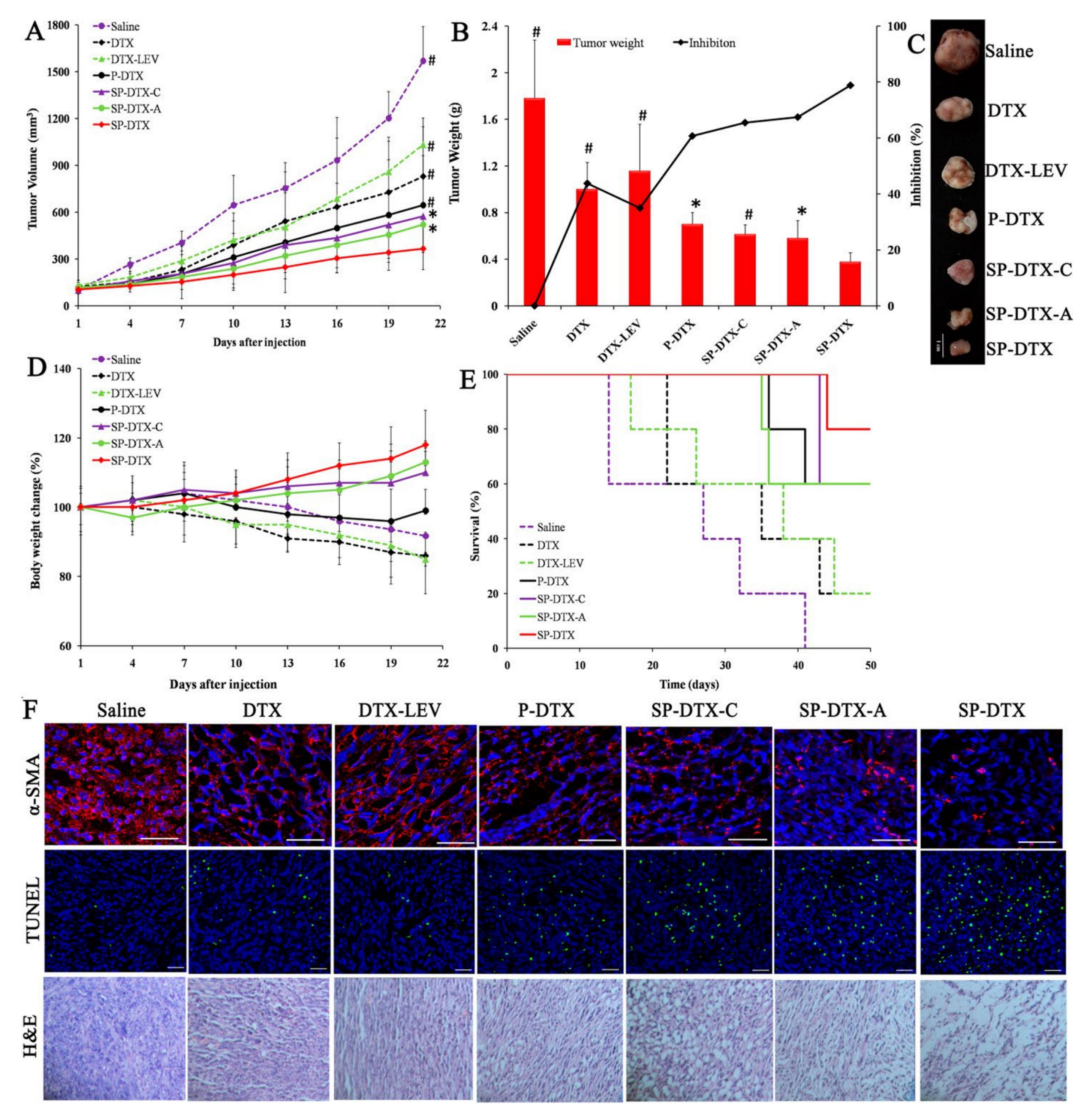
In vivo anticancer efficacy of various DTX formulations. (**A**) Tumor growth curves after intravenous injection of either saline, DTX, DTXLEV, P-DTX, SP-DTX-C, SP-DTX-A, or SP-DTX on mice bearing stroma-rich prostate xenograft tumors every 5 days for four times (5 mg/kg DTX). At the end of experiment, (**B**) isolated tumors were weighed and (**C**) photographed. (**D**) Body weight changes and (**E**) survival rates were monitored. (**F**) Effects of different treatments on the inhibition of CAF growth by α-SMA staining (red), the induction of apoptosis by TUNEL staining (green), and the histological examination by H&E staining of tumor tissues. Blue signal derived from nuclei stained by DAPI (*n* = 5, * *p* < 0.05, # *p* < 0.01 vs. SP-DTX). Reprinted with permission from ref. [92]. Copyright 2016 American Chemical Society.

Huang and co-workers synthesized a stimuli-responsive star-like POSS-based organic-inorganic conjugate, which self-assembled to afford nanoparticles with a high content of docetaxel (DTX) [92]. The authors grafted semitelechelic *N*-(2-hydroxypropyl) methacrylamide (HPMA) copolymers through a disulfide bond onto a rigid POSS core. POSS-based star polymer-drug conjugates were chemically loaded with DTX via a pH-responsive linker, which releases the drug under acidic/lysosomal conditions. DTX was added for physical encapsulation in the nanoparticles to enhance the loading of DTX, reducing the size of the nanoparticles to 57 nm due to the hydrophobic effect (Figure 1). The authors demonstrated the in vitro applications of this system using a PC-3 human prostate carcinoma cell line. DTX-POSS nanoparticles produce significant microtubule aggregation, G2-M phase arrest and cell apoptosis due to the effective release of DTX into the cytoplasm. To evaluate the efficacy of this DTX-POSS platform in vivo, the authors used a stroma-rich prostate xenograft tumor model. The tumor growth reduction results in this model showed that DTX-POSS nanoparticles could effectively improve the therapeutic efficacy of DTX as compared with control groups. Moreover, the survival rate analysis demonstrated that these nanoparticles effectively improved the survival of this drug regimen. Further analysis at the cellular level, showed that the platform’s enhanced antitumor efficacy was due to elimination of cancer-associated fibroblasts and induction of apoptosis (Figure 2).

Wu and his team have developed a POSS-based supramolecular AD (adamantine)-POSS-(sulfobetaine)7/CD (cyclodextrin)-PLLA (poly L-lactide) zwitterionic complex that takes advantage of the well-established host-guest chemistry between adamantine and cyclodextrin [34]. This stable complex can self-assemble into spherical nanoparticles, producing shell layers of zwitterionic supramolecular micelles where drugs such as doxorubicin (DOX) can be easily incorporated. The nanoparticles obtained by this approach are stable across a wide pH range and have resistance to protein adsorption. Moreover, by changing the pH in the solution, the controlled release of drug molecules can be achieved. The authors evaluated the cytotoxicity of POSS-based zwitterionic nanoparticles without and with DOX-loading. In the absence of DOX, the nanoparticles were fairly non-toxic up to 500 µg/mL in three different cell lines: MC3T3, MCF-7 and HeLa. However, a concentration-dependent cytotoxicity was observed in MCF-7 and HeLa cells when the POSS-based zwitterionic nanoparticles transport DOX.

Nair and co-workers synthesized POSS materials by applying the technique of hydrolytic co-condensation reactions. For that purpose, they mixed 3-aminopropyltriethoxysilane (AS) and vinyltriethoxysilane (VS) in an ethanol-water mixture and fabricated Pluronic F68 onto a POSS core [93]. Combining inorganic POSS and hydrophilic F68 ensures shape persistence and dispersibility in an aqueous medium through steric stabilization in the POSS-F68 hybrid vesicle. When doxorubicin (DOX) hydrochloride and folic acid (FOL) ligands were conjugated in the vesicle to form POSS-F68-DOX-FOL, it was expected that DOX would be distributed or adsorbed in the H-bonded form to the inner part of the vesicle wall and trapped inside the POSS cubes. As a result, the noncytotoxic FOL, when present in excess, will not interfere with cancer therapy. However, it could be H-bonded with the amino-propyl groups that are randomly present on the POSS-bilayer’s exterior surface. The authors demonstrated the controlled release behaviors of DOX, releasing approximately 40% of the encapsulated drug from the vesicle during seven days with no significant differences in phosphate buffer solution (PBS) at a body fluid pH of 7.4 and a slightly acidic pH of 6.9. They investigated in vitro cytotoxicity by measuring cell viability using MTT-assays and found their formulation to have major effects in L929 and HeLa cells following a 24 h incubation period with concentrations up to 1 mg mL^−1^.

Seifalian and co-workers developed a multifunctional nanoplatform. For that purpose, they grafted inorganic POSS with short polyethylene glycol chains (PEG), and anthracycline antibiotics called daunorubicin (DAU) to form soluble POSS-DAU and PEG-POSS-DAU conjugates [48]. The POSS cage can be activated by attachment of the anhydride rings and formed octakis-[3-(2-succinic anhydride) propyl, dimethylsiloxy] octa silsesquioxane (POSSAN), which reacts with daunorubicin or methoxy-PEG. Size analysis, optical absorption, and conjugate absorption spectrum confirmed their characteristic surface properties, sizes, and drug distribution in cells or tissues. HeLa cells are used to investigate in vitro anticancer cytotoxic activity. Therefore, the authors incubated the cells at five different concentrations (0, 5, 10, 50, 100 μM). They found an evident DAU dose-dependent cytotoxicity in the cells, where the cell viability decreased up to 40% at 50 μM concentration of drug in the presence of PEG4-POSS-DAU3 conjugates. The ability of the treatment to intercalate into DNA strands was confirmed by the gel electrophoresis method.

### 4.2. Gene Therapy

There are major challenges for the efficient transduction of genes through the cell membrane. Therefore, the need to develop DDS for gene delivery is present. POSS have been modified to render surface properties to allow interaction with the negatively charged backbone of DNA/RNA to afford nanomaterials capable of transporting and delivering genetic material inside of mammalian cells. Following this general approach, Feng and his coworkers designed biocompatible star/comb-shaped POSS copolymers by modifying a group of functional peptides. In their study, peptide-functionalized star-shaped copolymers self-assembled into nanoparticles (NPs), which condensed the pEGFP-ZNF580 (the recombinant plasmid of the enhanced green fluorescent protein plasmid) and rabbit anti-human ZNF580 polyclonal antibody (pDNA) to form NPs/pDNA complexes [39]. The presence of CAG (Cys-Ala-Gly) and TAT (Tyr-Gly-Arg-Lys-Lys-Arg-Arg-Gln-Arg-Arg-Arg)-NLS (Pro-Lys-Lys-Lys-Arg-Lys-Val) peptide sequences in the complex allowed high EC (endothelial cell) targeting and internalization capacity. The cationic PDMAEMA (poly (2-dimethylaminoethyl) methacrylate) also enhanced endosomal escape and blocked pDNA condensation, and hydrophilic PEG improved the biocompatibility. Thus, the complexes can exhibit higher cellular uptake, effective transfection, and expression of the genes carried by the pDNA into ECs. The authors assessed the in vitro cytotoxicity of the complexes in EA.hy926 cells treated with micelles or gene complexes formed at the N/P molar ratio of 15 for 48 h to determine cell viability. Moreover, they concluded that functional peptide-modified NPs and their gene complexes had low in vitro cytotoxicity, allowing them to act as a carrier for gene delivery.

**Figure 3:**
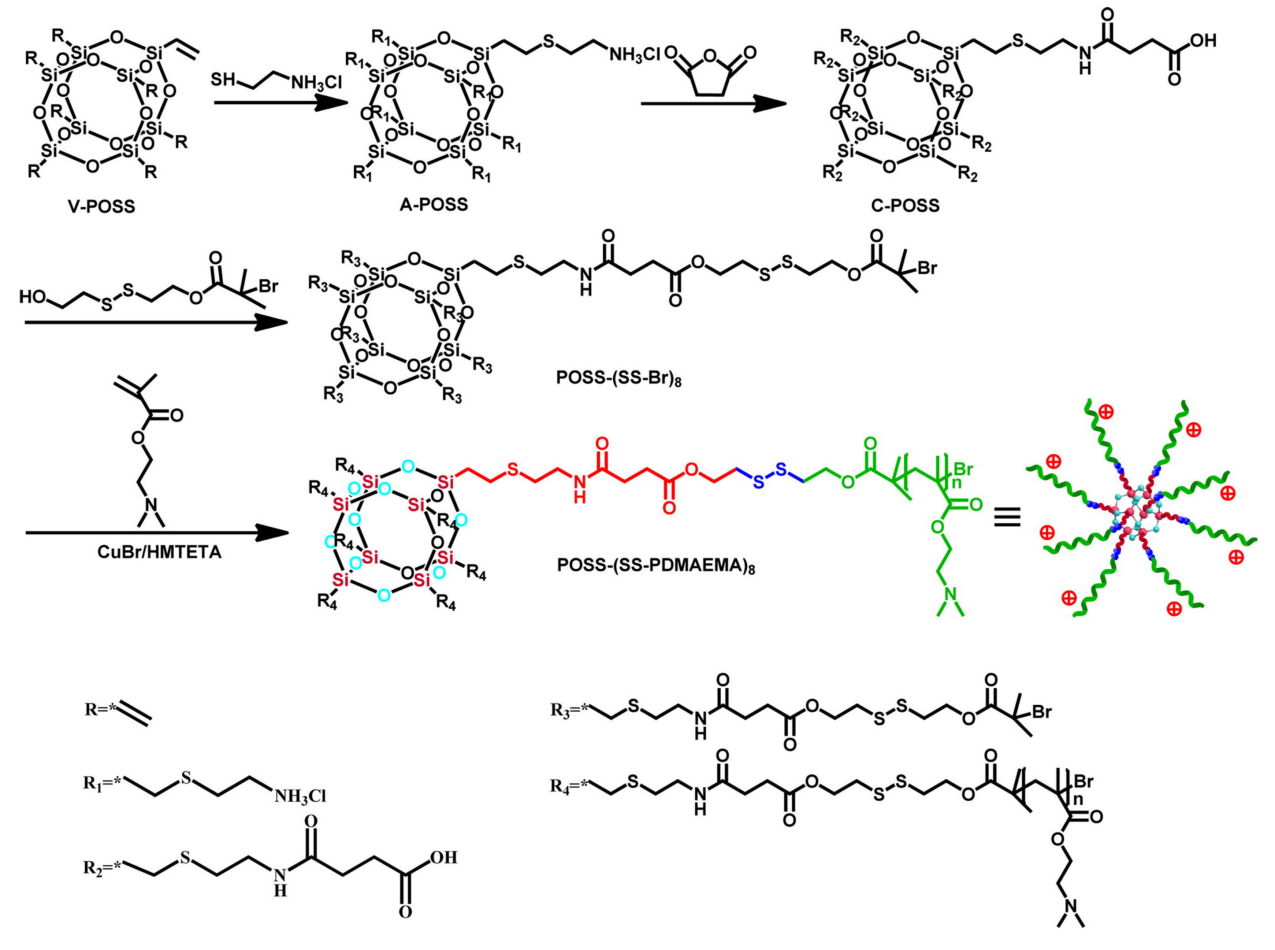
Synthetic processes of bioreducible POSS-based gene vectors via ATRP where * represents the POSS cage. Reprinted with permission from ref. [26]. Copyright 2014 American Chemical Society.

Xu and co-workers have also followed a similar type of technique. They demonstrated the synthesis of a bioreducible star-shaped gene vector (POSS-(SS-PDMAEMA)_8_) via an atom transfer radical polymerization (ATRP) method with (2-dimethylamino) ethyl methacrylate (DMAEMA) from a POSS macroinitiator (Figure 3) [26]. POSS-(SS-PDMAEMA)_8_ can effectively bind with pDNA into uniform nanocomplexes and improves transfection efficiency. This group assessed the cytotoxicity and gene transfection efficiency of nanocomplexes using HepG2 cells and COS7 cell lines using treatments with or without disulfide bonds. They concluded that disulfide bonds would ensure lower cytotoxicity and higher transfection efficiency in the system. Moreover, this technique can guide the researchers to design a POSS-based drug/gene carrier in the future.

### 4.3. Other Applications of POSS as DDS

Lukasz and co-workers have synthesized amido-functionalized compounds via benzoylation reaction between octa(3-aminopropyl) silsesquioxane hydrochloride (OAS-POSS-Cl) and an appropriate acyl chloride in the presence of NEt_3_ and N,N-dimethylformamide (DMF) [94]. This group used octa-functionalized amide-POSS as a carrier for non-steroidal anti-inflammatory drugs (NSAIDs) by trapping the drug molecules inside the platform, which was possible due to their nanosized architectural structures. In such a system, adsorbed drug molecules can be released under physiological conditions and the POSS-based carrier will be hydrolyzed (pH = 7.4) to a suitable non-toxic, carboxylic acid salt and a water soluble POSS containing an aminopropyl group, which can be safely removed from the organism.

Kim and co-workers have taken advantage of novel nanostructured core-shell poly (ethylene glycol) (PEG)-POSS nanoparticles. The team utilized these nanoparticles to encapsulate insulin as a drug delivery carrier. This article demonstrates via TEM analysis that pure and insulin-loaded self-assembled PEG-POSS nanoparticles are spherically shaped with core-shell nanostructures, well-dispersed, and have uniform size distribution. The insulin release occurred at intestinal pH (pH 6–7), checked via an insulin release test from well-protected PEG-POSS nanoparticles at gastric pH for 2 h [95].

## 5. Applications of POSS in Photodynamic Therapy

PDT is a non-invasive therapeutic modality that relies on three main components: oxygen, light and a photosensitizer agent. Photosensitizers are usually hydrophobic molecules which require DDS for their effective transport and delivery on the target tissue. Silica-based nanoplatforms such as MSNs and PSilQ NPs have been used for that purpose [90,91,96,97]. The POSS platform is an attractive alternative approach for the delivery of photosensitizers because POSS can be modified with multiple functional groups to enhance their solubility and render targetability. In addition, POSS derivatives can easily be incorporated into polymers, adding functional groups into the polymer matrix to render unique self-assembly properties. Interestingly, the relatively large molecular volume of the POSS can provide steric hindrance to prevent the aggregation of photosensitizers, which results in the decrease of singlet oxygen generation efficiency due to the aggregation-induced quenching effect (AIQE). Below we illustrate some of the most recent examples of the use of POSS for PDT.

### 5.1. Multifunctionalization

**Figure 4:**
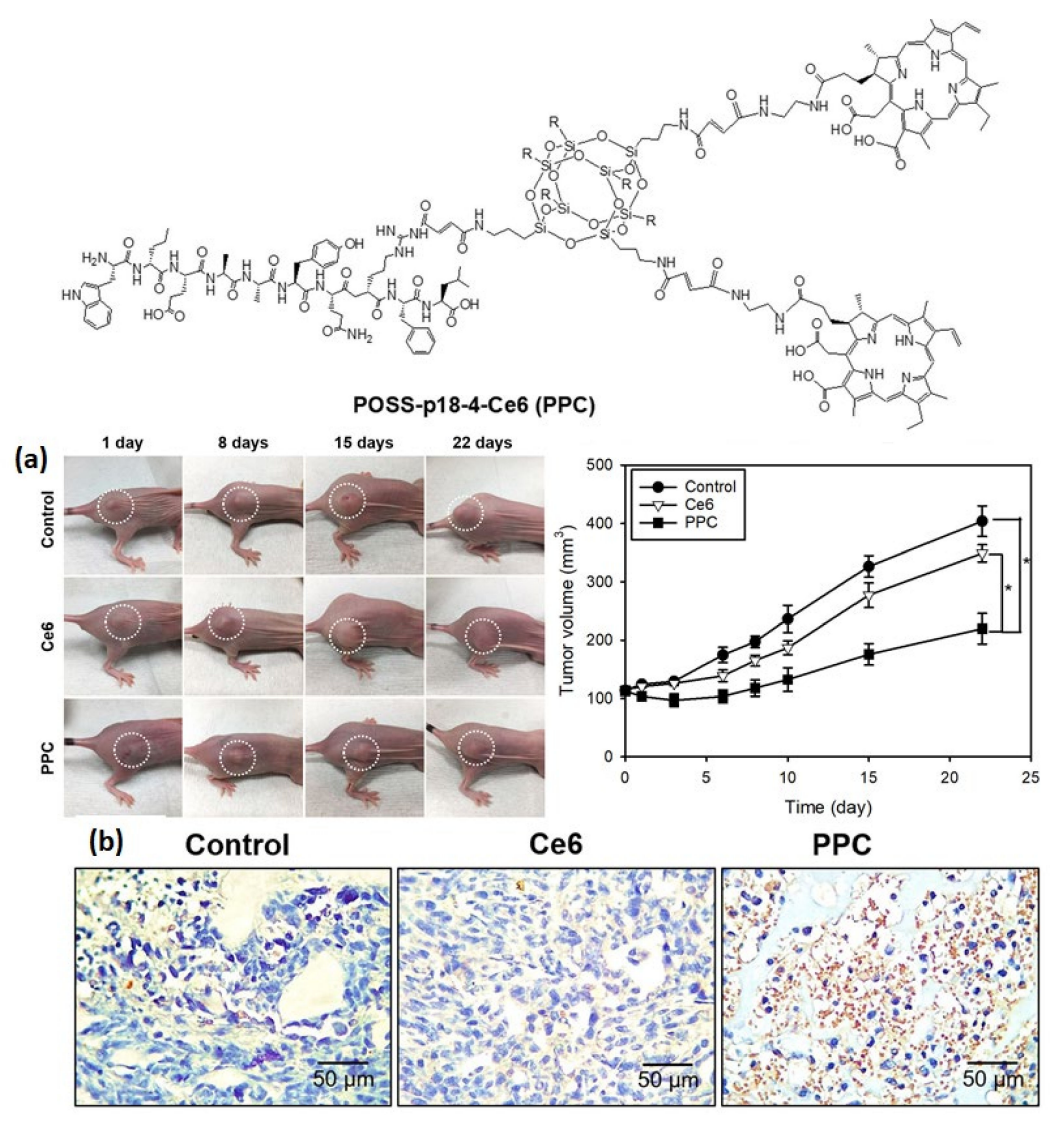
p 18-4/Ce6-conjugated POSS (PPC) nanoparticles. In vivo PDT efficacy of PPC nanoparticles (*n* = 4). (**a**) Tumor images and (**b**) quantitative analysis of tumor growth after PDT with free Ce6 or PPC nanoparticles in tumor-bearing mice. Differences between groups were tested using one-way ANOVA. * *p* ≤ 0.05. Histological TUNEL staining for tumor slices harvested from tumor-bearing mice after 22 days of PDT with free Ce6 or PPC nanoparticles. Reprinted with permission from ref. [55]. Copyright 2020 Elsevier.

In addition to carrying the photosensitizer, POSS molecules have been further functionalized with other molecules such as targeting agents. Kim and co-workers attached chlorin e6 (Ce6) moieties to octamalemic acid POSS and peptide 18-4, which targets specifically breast cancer cells (Figure 4) [55]. The peptide-targeted Ce6-POSS molecules self-assemble in water into nanoparticles smaller than 200 nm in diameter, which is adequate for cell uptake and in vivo applications. The cellular uptake and phototoxicity of the nanoparticles were enhanced in MDA-MB-231 breast cancer cells with respect to the free photosensitizer and other cancer cell lines due to the presence of the targeting peptide. The peptide-targeted Ce6-POSS nanoparticles were accumulated effectively in the tumor tissue in a MDA-MB-231 tumor-bearing mouse model. These nanoparticles also showed an improved phototherapeutic effect in vivo resulting in a decrease in tumor size and a significant increase in the number of apoptotic cells in the tumor tissue (Figure 4). The use of POSS as a building block in this work afforded nanoparticles with increased generation of ROS, which effectively increased PDT efficacy in vitro and in vivo.

**Figure 5:**
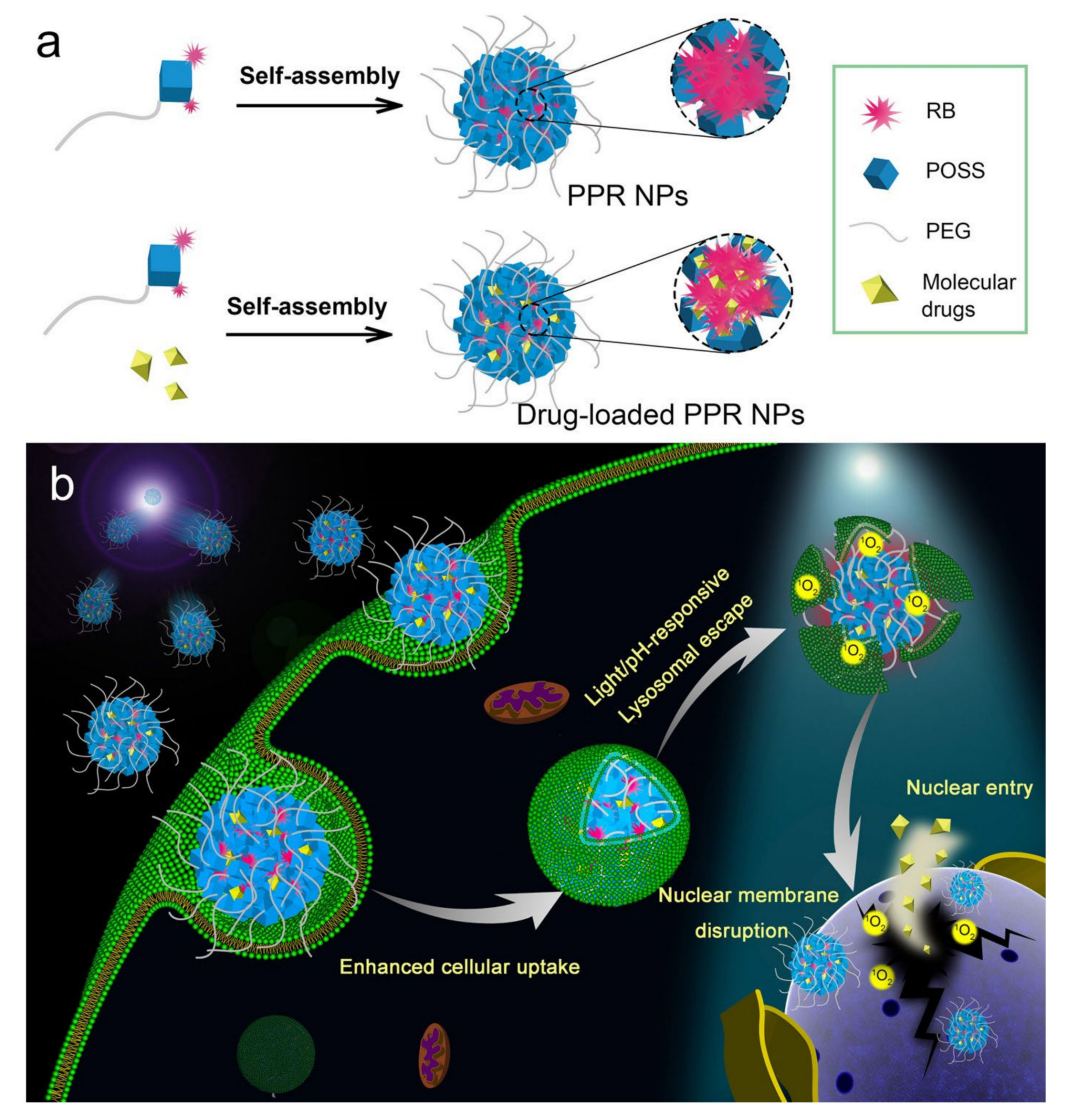
(**a**) The formation of PPR NPs and PPR/HCPT NPs and (**b**) the pH-responsive and light-triggerable nuclear delivery strategy using PPR/HCPT NPs as an example. Reprinted with permission from ref. [98]. Copyright 2018 American Chemical Society.

Nuclear membrane and nuclear pore complexes (NPCs) are a barrier for nucleus targeting drugs like 10-hydroxycamptothecin (HCPT) and docetaxel (DTX). Fu-Gen et al., offer a platform that consists of a polyamine-containing polyhedral oligomeric silsesquioxane (POSS) unit, a hydrophilic polyethylene glycol (PEG) chain, and the photosensitizer rose bengal (RB) that can self-assemble into nanoparticles (denoted as PPR NPs) (Figure 5) [98]. When PPR NPs are loaded with drugs and irradiated with mild light, increased singlet oxygen (^1^O_2_) is generated in response to lysosomal acidic conditions, and efficient lysosomal escape occurs via the photochemical disruption of lysosomes. This team has incubated A549 cells with free HCPT or PPR/HCPT NPs at different incubation periods to investigate their cell internalization capacity to the cancer cells and several analytical techniques to observe the consequences after nuclear entry. They concluded that the released nanoparticles could accumulate on nuclear membranes and augment membrane permeability by inducing lipid peroxidation upon further light exposure.

Fu-Gen and group members have crosslinked a carboxyl-containing photosensitizer, Chlorin e6, into an amine-containing POSS structure. An amine-carboxyl reaction forms a 3D network via 1-ethyl-3-(3-dimethyl aminopropyl)-carbodiimide (EDC)/N-hydroxysuccinimide (NHS) coupling method. The resulting POSS-Ce6 shows an improved dispersibility in aqueous media and prolonged circulation time after PEG coating. The final nano agent (POSS-Ce6-PEG) provides a high degree of chemical stability in PDT with significant drug-loading capacity [45].

Lee and Kim conjugated Protoporphyrin IX (PpIX) molecules and linolenic acid to a water soluble octammonium POSS. The molecules self-assembled into nanoparticles of ~120 nm in diameter due to strong hydrophobic interactions [56]. Cellular uptake was enhanced, most likely due to the presence of linoleic acid. The nanoparticles also showed improvement in the in vitro PDT efficacy, while dark toxicity was reduced compared with PpIX. The cellular death mechanism was shifted mainly from necrosis, which is related to PpIX to apoptosis with the nanoparticles. This shift in the mechanism is beneficial for therapy since necrosis usually results in inflammation.

Zhang and co-workers synthesized a unimolecular micelle based on an amphiphilic star shaped block copolymer, which is made of hydrophobic poly(E-caprolactone)(PCL) and hydrophilic poly(2-(dimethylamino)ethyl methacrylate) (PDMAEMA). The photosensitizer pheophorbide A was loaded in the hydrophobic portion in the inner layer of the micelle [99]. Additionally, the hydrophilic outer layer (PDMAEMA) was further functionalized with biotin as a targeting agent. The micelles depicted a pH-sensitive release of pheophorbide A under acidic conditions. The micellar system resulted in increased uptake by HeLa cells and an enhanced PDT efficiency with low dark toxicity compared with the free photosensitizer.

### 5.2. Use of POSS to Prevent Aggregation-Induced Quenching Effect

Jin and co-workers incorporated pendant isobutyl POSS monomers into an amphiphilic copolymer that alternates a tetraphenyl porphyrin with the POSS. With this arrangement, the authors prevented porphyrin aggregation and reduced the AIQE [100]. The system then self-assembled into spherical nanoparticles with sizes dependent on the polymer length. Compared to the POSS-free polymeric nanoparticle, this system showed a higher fluorescence and singlet oxygen quantum yield as a clear indication that the POSS units contribute to the decrease of the aggregation between porphyrin units. In vitro data in A549 cells showed the time-dependent internalization of the nanoparticles and the enhanced PDT efficiency associated with the POSS units. Finally, the POSS containing polymeric nanoparticles have excellent biocompatibility and anti-cancer capability for the PDT of tumors in vivo.

**Figure 6:**
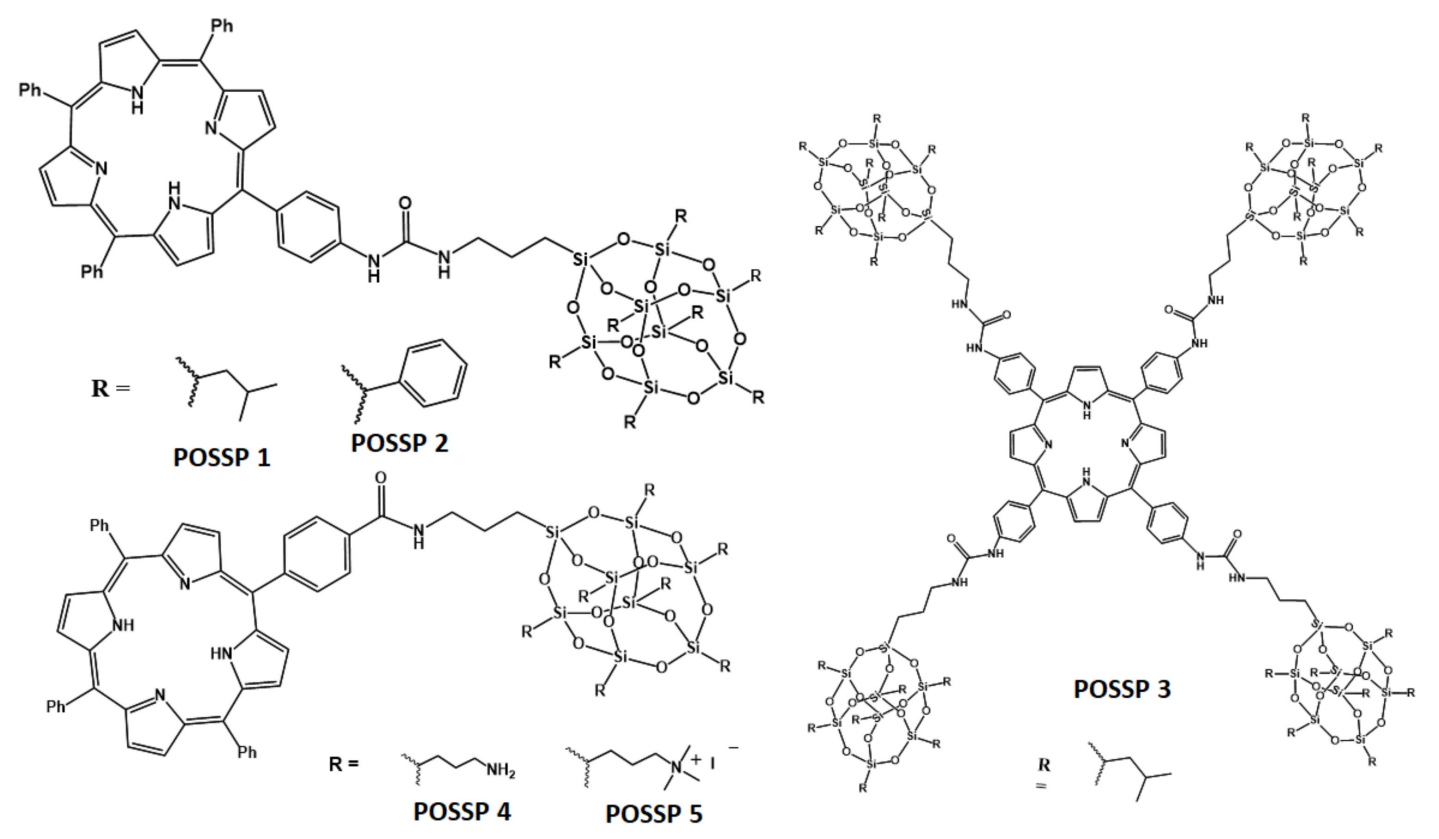
POSSP derivatives which were synthesized in [27]. POSSPs 1–3 contain hydrophobic groups. POSSPs 4 and 5 are functionalized with hydrophilic moieties.

Our group has also functionalized POSS molecules containing different substituents linked to a porphyrin photosensitizer [27]. A series of five POSS-porphyrin derivatives were synthesized containing different types of moieties in the POSS cage (Figure 6). We found that the substituents in the POSS molecule affect the aggregation of the porphyrin unit differently. Hydrophobic substituents like isobutyl showed a better performance to prevent the aggregation of the porphyrin unit. This steric effect increased singlet oxygen quantum yield. The in vitro properties of the POSS-porphyrin compounds were evaluated in a triple-negative breast cancer cell line (MDA-MB-231). Interestingly, the molecules with phenyl substituents on the POSS showed a better cellular uptake than the hydrophilic ones. Nevertheless, due to the enhanced singlet oxygen quantum yield, POSS-porphyrin molecules with hydrophobic substituents showed the highest phototoxicity. Molecular dynamics (MD) simulations were used to study the aggregation of POSSP-1, POSSP-2, and POSSP-3 [101]. The MD results show that the hydrophobic effect was driving the self-assembly of POSSP-1 and POSSP-3, resulting in aggregates where the porphyrins were apart from each other. The decrease of the AIQE accounts for the improved PDT performance of these POSS-porphyrin derivatives.

**Figure 7:**
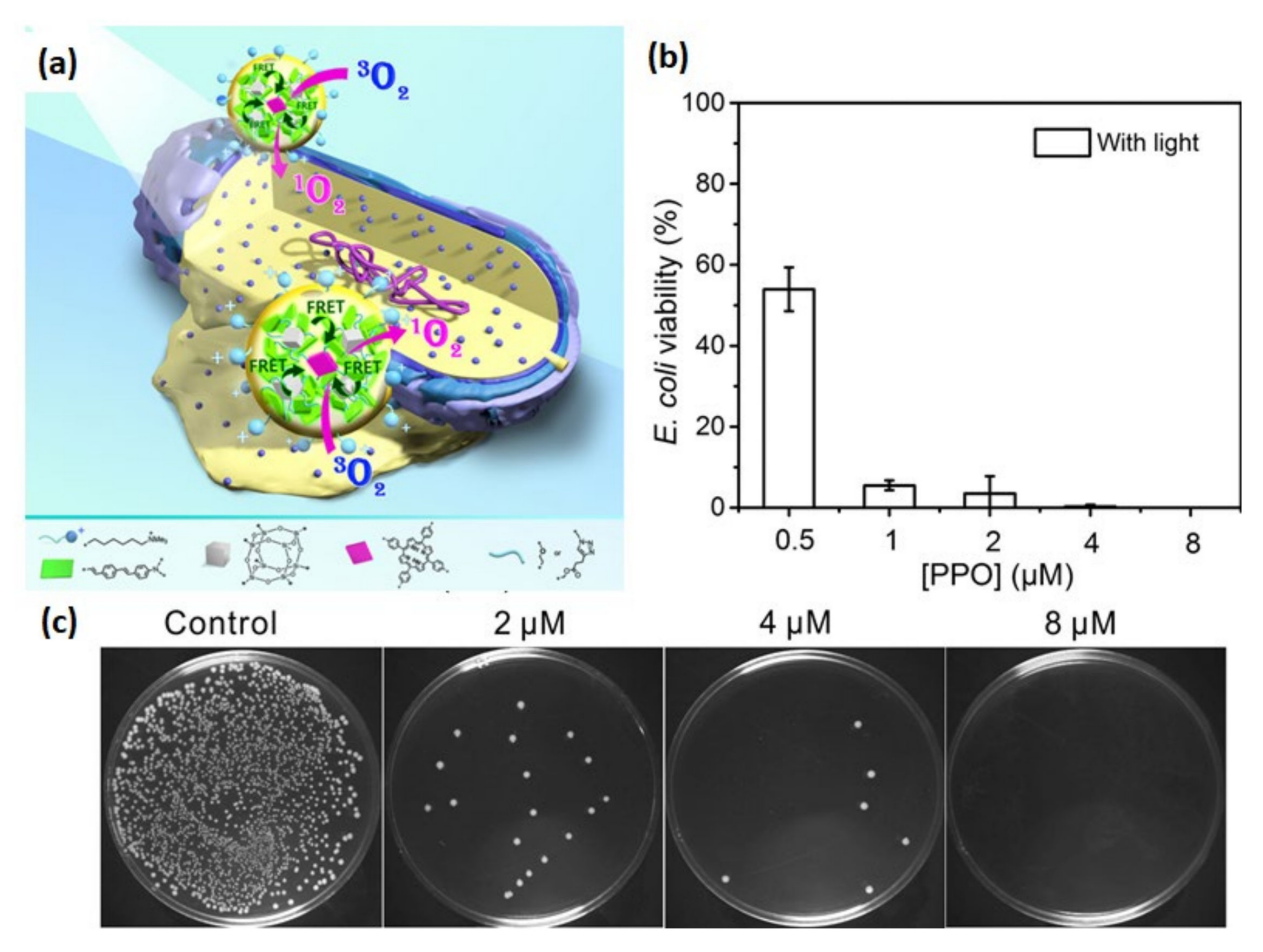
(**a**) Schematic representation of the design and work principle of POSS-porphyrin system. (**b**) Percentage of *E. coli* viability after treatment with a series of concentrations of PPO under irradiation (12 mW·cm^–2^, 10 min). (**c**) Representative photographs of lysogeny broth (LB) agar plates for *E. coli* treated with a series of concentrations of PPO under white light irradiation. Reprinted with permission from ref. [96]. Copyright 2018 American Chemical Society.

Bao, Wang and co-workers modified POSS units with cationic conjugated oligoelectrolyte pendants containing terminal quaternary ammonium groups [96]. These cationic POSS molecules were further used to functionalize a central tetraphenyl porphyrin. The POSS arms contain oligo(p-phenylenevinylene), a light-harvesting antenna that can transfer its excitation energy to a porphyrin, enhancing its photophysical properties such as singlet oxygen production (Figure 7a). The quaternary ammonium groups on the POSS units accomplish two critical roles; they prevent the aggregation of the system in aqueous media and increase the electrostatic interaction with bacterial membranes. Because of the presence of the cationic oligo(p-phenylenevinylene) arms, singlet oxygen generation was increased more than four times with respect to the control porphyrin, which confirms the efficient energy transfer to the porphyrin core. The POSS-porphyrin system showed dark toxicity against Gram-negative bacteria (*E. coli*) due to its high positive charge density. Moreover, under white light irradiation, the POSS-porphyrin platform eliminated 99.9% of Gram-negative *E. coli* or Gram-positive *S. aureus* using 8 μM or 500 nM, respectively (Figure 7b,c). The system exhibited combinatorial antibacterial efficacy due to the presence of POSS that prevented aggregation, enhanced singlet oxygen generation, and increased the local cationic density to disrupt the bacterial membrane.

The same group also modified tetrahydroxyphenyl porphyrin with POSS units containing long dodecyl alkyl chains. With this strategy, the authors expected to overcome the lack of water solubility and aggregation of the photosensitizers [50]. In addition, the authors also wanted to amplify the singlet oxygen generation by energy transfer after the POSS-porphyrin unit was wrapped by a semiconducting polymer forming nanoparticles. Photophysical characterization of the nanoparticles showed an improved fluorescence quantum yield and singlet oxygen generation as an indication that the POSS scaffold and the long alkyl chains effectively reduces the aggregation of porphyrins and prevents the interaction with the semiconducting polymer. In vitro evaluation of the nanoparticles in HeLa cells demonstrated an improved fluorescence emission in biological media and the PDT effect.

**Figure 8:**
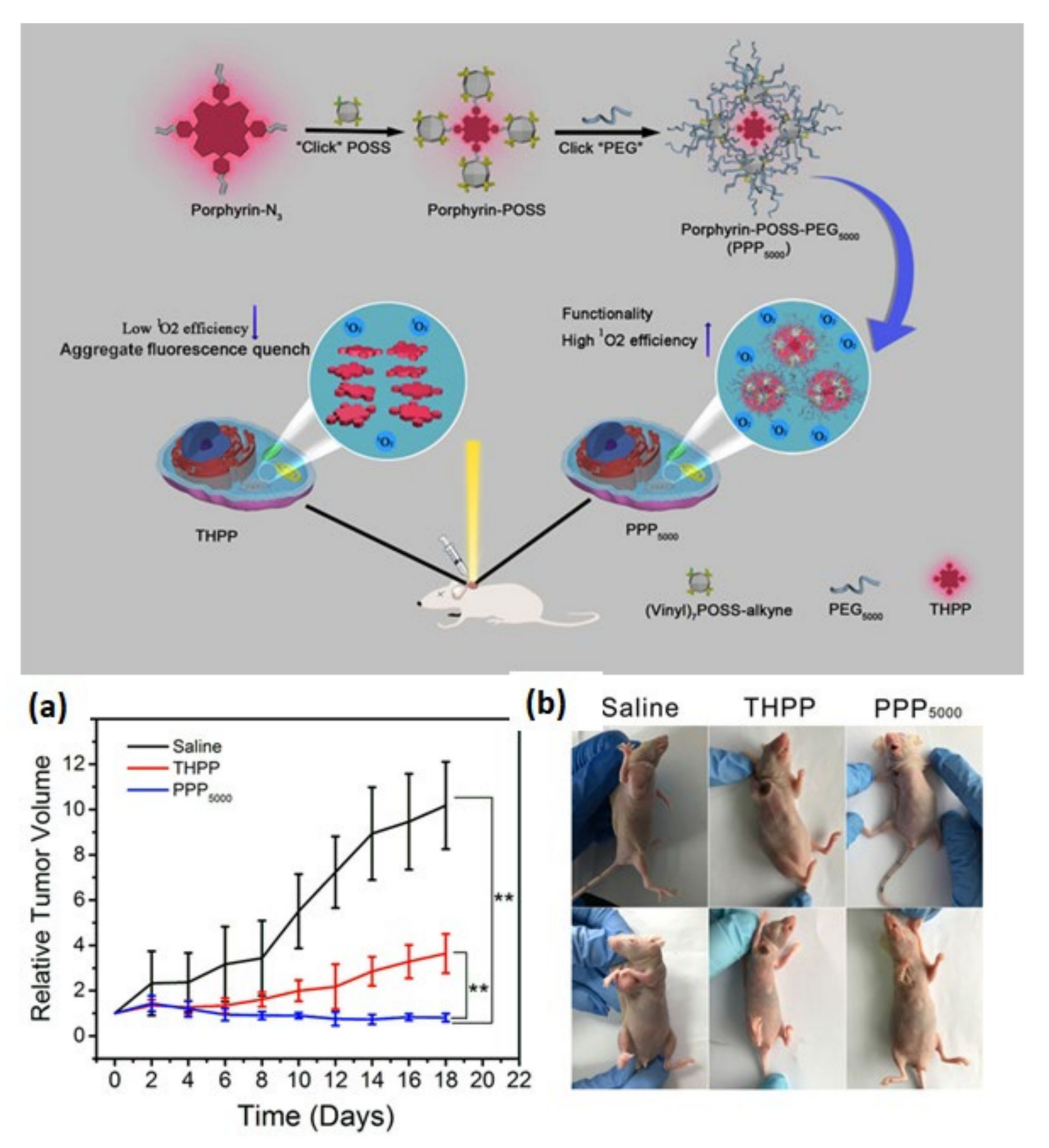
Schematic illustration of the fabrication process and photodynamic therapy of PPP5000 and THPP. (**a**) Changes of relative tumor volume (V/V_0_) after mice were treated with saline, THPP, and PPP5000. Differences between groups were tested using one-way ANOVA. ** *p* ≤ 0.01. (**b**) Representative photos of mice after treatment. Reprinted with permission from ref. [49]. Copyright 2018 American Chemical Society.

Bao, Wang and co-workers conjugated four POSS to a tetrahydroxyphenyl porphyrin (THPP) where the POSS arms were functionalized with PEG5000 to create water-soluble nanoparticles with a hydrodynamic diameter of 28 nm (Figure 8) [49]. The fluorescence and singlet oxygen generation were enhanced with respect to the control porphyrin due to the presence of the POSS framework, which reduced the self-quenching effect between porphyrins. In vitro tests on HeLa cells showed lower dark toxicity but enhanced toxicity under irradiation as compared with THPP. The primary mechanism of cell death is via apoptosis. In in vivo PDT tests, the nanoparticles were more effective than the free porphyrin, achieving complete ablation of tumor tissue (Figure 8). Overall, these data demonstrate that the nanoparticle has a better PDT efficacy than THPP associated with the anti-aggregation and biocompatibility of POSS scaffolds and PEG branches.

Wu and co-workers used octaaminopropyl POSS as a building block to fabricate a nanoparticle which contains a network of crosslinked Ce6 photosensitizers [45]. The resulting POSS-Ce6 nanoparticle was further functionalized with PEG, affording a nanoconstruct 70 nm in diameter with high stability in physiological conditions. This approach allowed a high Ce6 loading of 19.8 wt%. No major changes in fluorescence and a slightly reduced amount of singlet oxygen generation were observed compared with free Ce6. The fact that the POSS-Ce6-PEG nanoparticles maintained the photophysical properties of Ce6 was somewhat surprising considering the amount of Ce6 that had been encapsulated. This result is related to the steric effect associated with the POSS building blocks. In vitro experiments demonstrated that POSS-Ce6-PEG has a higher cellular uptake and PDT effect in HeLa cells relative to free Ce6. Confocal microscopy showed that the nanoparticles were mainly localized in the ER and mitochondria. In vivo evaluation of the nanoplatform in a xenograft mouse model depicted a higher accumulation in tumor tissue relative to the free Ce6 photosensitizer, most likely due to the EPR effect. Moreover, no dark toxicity was detected with these nanoparticles, which were cleared from the body a few days after intravenous injection. PDT efficacy in this animal model showed that the POSS-Ce6-PEG nanoparticles completely ablated the tumor.

### 5.3. Other Uses of POSS for PDT

Zhang and co-workers utilized POSS as a template for the fabrication of hollow nanoparticles. The system was synthesized using an amphiphilic block copolymer containing the light/redox-responsive monomer coumarin methacrylate, the pH-responsive hydrophilic monomer 2-(dimethyl amino) methacrylate, and an isobutyl POSS based methacrylate monomer [97]. This polymer self-assembled in an aqueous solution into 250 nm spherical micelles with the POSS in their core. The POSS template was removed by etching with HF, affording pH-responsive hollow nanocapsules 200 nm in diameter. Tetraphenylporphyrin tetrasulfonic acid hydrate (TPPS) was used as a photosensitizer to evaluate the use of these hollow nanocapsules for PDT. TPPS was efficiently loaded, and under reducing and low pH conditions, such as those found in the intracellular environment of tumor cells, the capsules disintegrated to release the porphyrin. In vitro experiments showed that these nanocapsules facilitated the internalization of TPPS in MCF-7 cells, most likely through an endocytic pathway. Conversely, the free TPPS is internalized only by a passive mechanism. Phototoxic experiments confirmed that the nanocapsules are more efficient than the parent porphyrin.

## 6. Applications of POSS in Bioimaging

In recent decades, bioimaging has attracted much attention due to its ability to obtain anatomical and physiological details of bio-systems ranging from cell to ex vivo tissue samples and in vivo imaging of living objects [102]. Bioimaging techniques such as fluorescence, magnetic resonance imaging (MRI), and X-ray tomography (CT) can label targeted objects and prepare information of the anatomic structure of tissues. In comparison with conventional biological labels such as organic dyes, nanotechnology possesses superior physicochemical features, including low auto-fluorescence, large anti-Stokes shifts, low toxicity, high resistance to photobleaching, and high penetration depth [103,104]. The use of POSS for bioimaging applications has been focused on two main directions: as delivery systems for molecular contrast agents, or as coating agents for nanoprobes. In both cases, the distinctive advantages of POSS: easy functionalization, chemical stability, and biocompatibility have allowed researchers to develop contrast imaging platforms with improved water stability, low toxicity and imaging capabilities.

### 6.1. POSS for Delivery of Contrast Agents

Rotello and coworkers pioneered the use of POSS for bioimaging applications. They reported on the modification of octaammonium-POSS (OA-POSS) with boron-dipyrromethene (BODIPY) as a cellular fluorescent marker [43]. The POSS-BODIPY molecules showed excellent solubility in an aqueous environment due to the overall positive charge associated with the free ammonium groups in OA-POSS. In vitro evaluation in Cos-1 cells showed that POSS-BODIPY is non-cytotoxic at concentrations as high as 1 mM. In addition, fluorescence confocal microscopy images demonstrated the efficient internalization of POSS-BODIPY and co-localization in the cytosol.

Inspired by Rotello’s reports on the use of POSS for bioimaging applications, Marchese and collaborators prepared a novel, highly luminescent bifunctional POSS containing its their structure a fluorescein derivative (FITC) as a fluorophore (POSS_F) and a carboxylic functionality ready to anchor several organic/inorganic molecules for biomedical applications [31]. The structural features of POSS_F were fully characterized by infrared (IR) spectroscopy, ^1^H and ^29^Si NMR, and mass spectrometry. The light absorption properties of POSS_F are similar to the parent fluorophore. However, the emission intensity of POSS_F increased approximately four times compared to the corresponding equimolar FITC solution. The authors evaluated the in vitro performance of POSS_F using fluorescence and confocal microscopy in HeLa cells. The microscopy data suggested that the migration of the POSS_F molecules to the cell cytoplasm was time-dependent and driven by the high affinity of the POSS to the membrane double layer of HeLa cells. In addition, inhibition experiments of different endocytic pathways indicated that POSS_F molecules are endocytosed via the macropinocytosis route.

Miksa and coworkers have developed biocompatible POSS-based phenosafranin dye (PSF) nanohybrids using a coupling chemistry for drug delivery and diagnostics [105]. PSF is used as a photosensitizer, and a biological probe acts as a nucleic acid intercalator and human ribonuclease reductase inhibitor. POSS-PF afforded fluorescent nanodots with cationic charges on the surface, which are easily permeable through cellular pores and can be useful for cellular imaging and cancer treatment. In vitro experiments using HeLa cells revealed that the platform is non-cytotoxic and can be easily internalized by cells via diffusion rather than endocytosis. Preliminary data in this paper also provided evidence that POSS-PSF intercalates DNA. Therefore, it is envisioned that the platform can be used for anticancer therapy.

**Figure 9:**
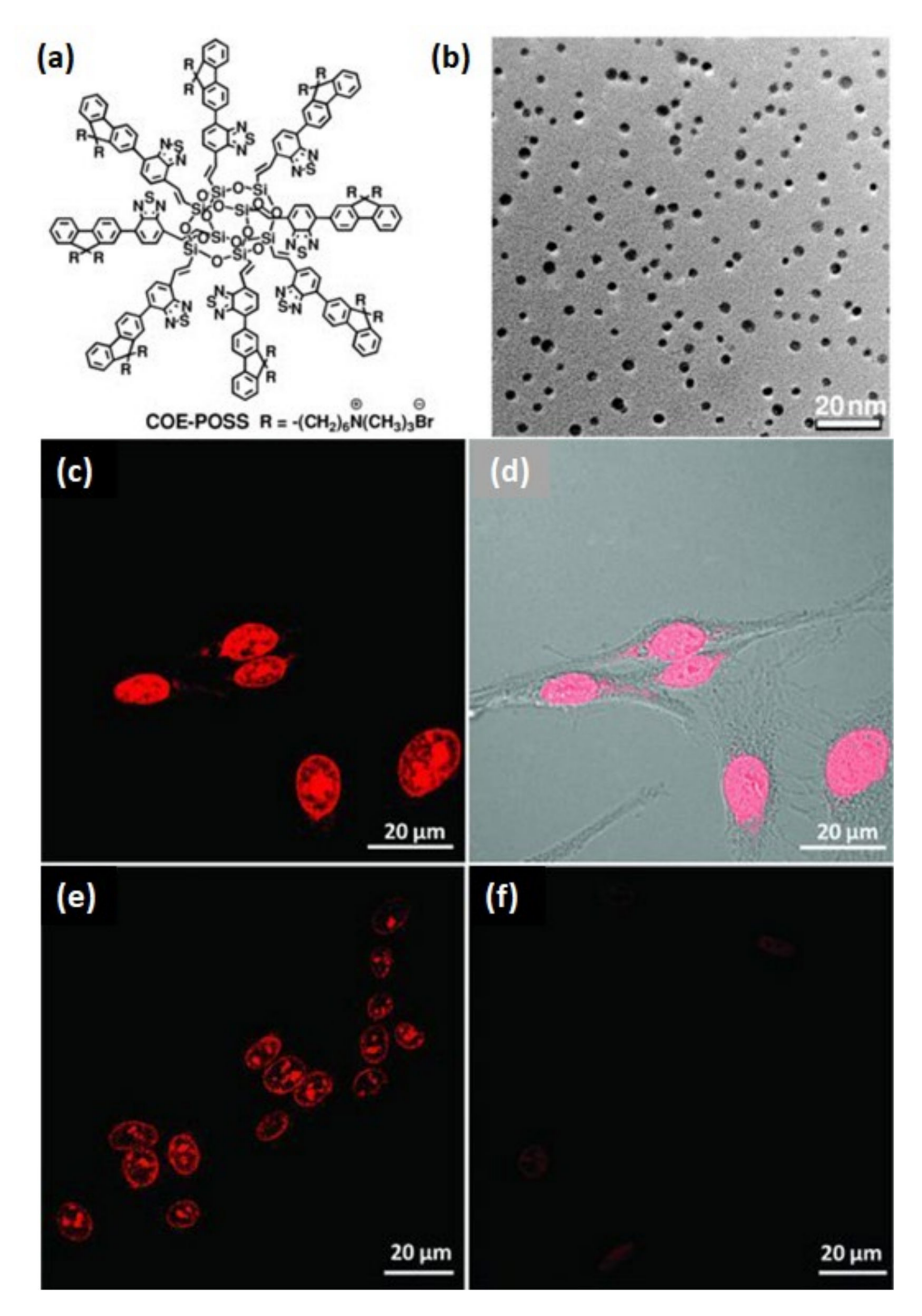
(**a**) Chemical structure and (**b**) HR-TEM image of COE-POSS. (**c**) OPEF and (**d**) OPEF/transmission overlapped images of MCF-cells stained with 1 μM COE-POSS. The signals are collected above 560 nm upon excitation at 488 nm. TPEF images of MCF-7 cells incubated with 1 μM COE-POSS (**e**) or SG (**f**) for 2 h. Reprinted with permission from ref. [62]. Copyright 2010 John Wiley and Sons.

Liu et al. reported on the development of a three-dimensional, water-soluble, hybrid nanodot based on POSS and a conjugated oligoelectrolyte (COE) for two-photon excited fluorescence (TPEF) (Figure 9) [62]. In particular, the authors were interested in developing an efficient nanoprobe for TPEF imaging of the cellular nucleus. The COE-POSS was synthesized using the Heck coupling reaction. Despite the relatively low quantum yield of COE-POSS in water, once the platform is associated with RNA/DNA, it formed tight complexes through electrostatic attractions, which resulted in a dramatic increase of the quantum yield, photoluminescence (PL) and two-photon absorption (TPA) cross-sections. It is important to point out that the particle size of COE-POSS is 3.3 nm and well within the effective transportation diameter of the nuclear pore complex (~9 nm). The authors evaluated the TPEF imaging capability of this nanoprobe in breast cancer cells (MCF-7) as a proof of concept. COE-POSS exhibited low cytotoxicity and efficient nucleus permeability. TPEF micrographs of COE-POSS depicted a strong fluorescence from the nuclei due to the presence of a large amount of DNA/RNA. The performance of COE-POSS for the TPEF imaging of the cellular nucleus was superior compared to SYBR Green I (SG), which is one of the most sensitive commercially available dsDNA stains. Therefore, this new platform based on POSS holds excellent potential as a contrast bioimaging agent for clinical diagnosis and modern biological research.

### 6.2. POSS as a Coating Agent for Nanoprobes

**Figure 10:**
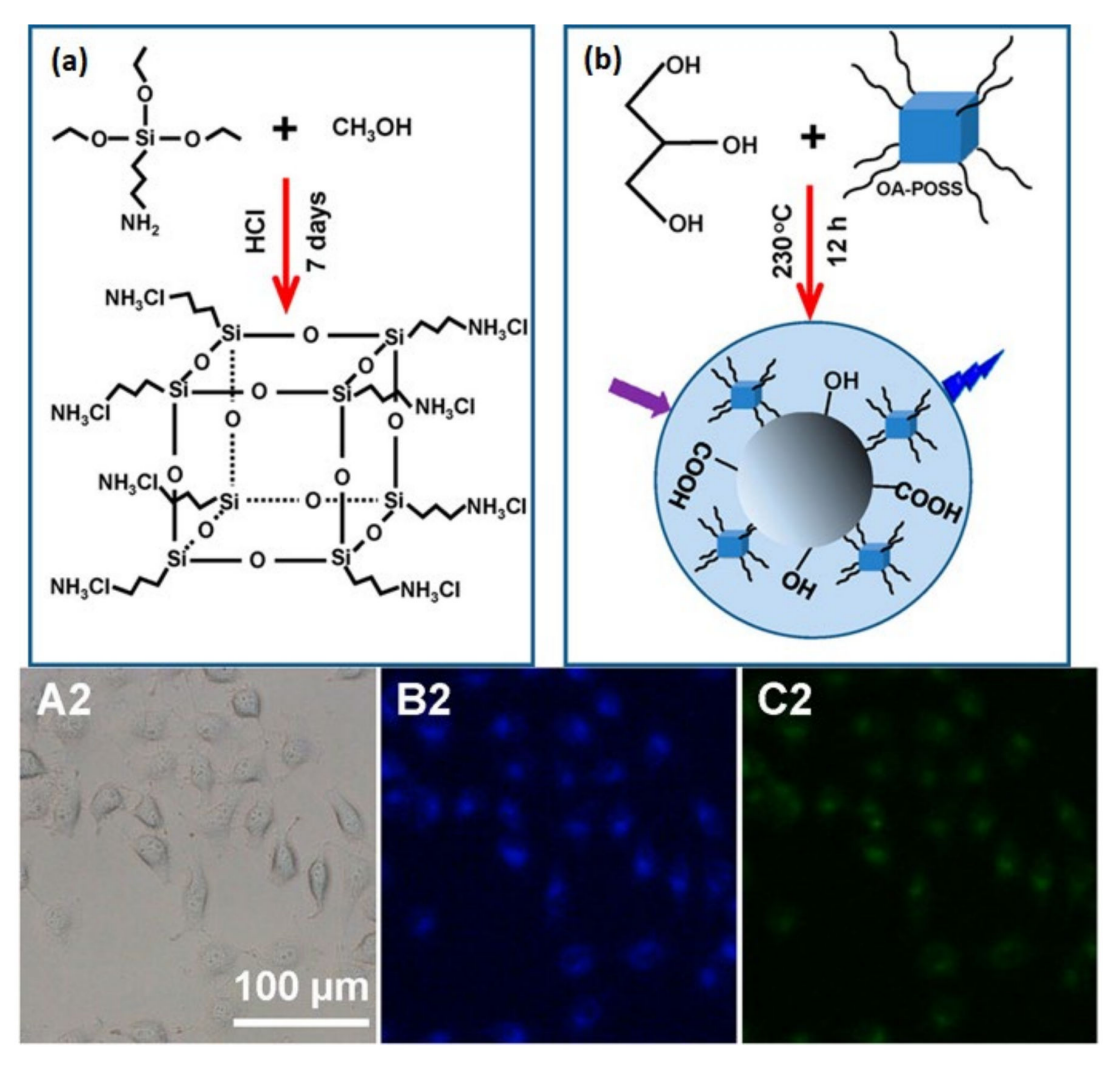
(**a**) Illustration for the hydrolytic condensation of APTES to produce OA-POSS and (**b**) preparation of carbon dots (CDs/POSS) with glycerol as carbon source and OA-POSS as passivation agent. Fluorescent microscope images of (**A2**–**C2**) MCF-7 cells labeled with CDs/POSS. (**A2**) Bright-field images; (**B2**) with an excitation wavelength of 340 nm; (**C2**) with an excitation wavelength at 495 nm. Reprinted with permission from ref. [42]. Copyright 2015 American Chemical Society.

Wang and his group have explored the use of octaammonium-POSS (OA-POSS) to functionalize the surface of carbon dots (CDs). Bare CDs without surface functionalization or passivation generally exhibit very weak emissions in aqueous medium and other solvents. The passivation is carried out through the electrostatic interaction between the ammonium groups of OA-POSS and the carboxylate groups on the surface of CDs (Figure 10). As a result, organic-inorganic hybrid CDs with a diameter ca. 3.6 nm can be obtained and well dispersed in the aqueous medium [42]. High quantum yield, resistance to photobleaching, and excellent photoluminescence stability are achieved by passivating CDs with OA-POSS. In vitro evaluation in HeLa cells using the MTT assay showed that CDs/POSS nanoparticles are biocompatible and can be used for multicolor cell imaging.

Rare earth-doped upconversion nanoparticles (UCNPs) are considered a new promising generation of imaging agents for bioimaging [106,107]. UCNPs have unique features, including resistance to photobleaching and low background autofluorescence, excellent biocompatibility, low cytotoxicity, and water stability. Usually, UCNPs need to be modified to add hydrophilicity for successful application in the biological field [108]. Nevertheless, current functionalization strategies have some limitations in the forms of complicated synthesis processes or post-treatment procedures, fluorescence quenching, and aggregation of nanoparticles. Therefore, there is a need to develop more straightforward surface modification methods of UCNPs. To this end, Chen and his group have explored POSS as a multifunctional coating agent for UCNPs. The authors reported on the synthesis of POSS-UCNPs(Er) and POSS-UCNPs(Tm) obtained after functionalization with POSS of NaYF4:Yb,Er@NaGdF4 and NaYF4:Yb,Tm@NaGdF4, respectively [109]. Photophysical and in vitro characterization showed that these POSS-UCNP materials have excellent upconversion luminescence (UCL), photostability, stability in biological media, and biocompatibility. The authors further evaluated the in vivo toxicity of the POSS-UCNP platforms in Kunming mice. Data analysis of the hematoxylin and eosin (H&E)-stained tissue sections of main organs (heart, lung, liver, spleen, and kidney) and serum biochemistry assays specific to potential risks of injury to liver, spleen, and kidney indicated that POSS-UCNPs are safe in the dose range reported in this study. The authors successfully tested the imaging performance of the POSS-UCNPs for both MRI and UCL imaging in vivo. MRI images showed enhanced MR signals in the liver and spleen as an indication that these nanoparticles could circulate in the liver in a short time. Similarly, a significant UCL signal in vivo was observed with high contrast compared with the background after intravenous injection in a nude mouse. The in vivo imaging results showed that the POSS-UCNPs can serve as promising contrast agents for in vivo MRI and UCL imaging. Overall, the method of POSS modification provides POSS-UCNPs with colloidal stability, excellent biocompatibility, and good MR/UCL imaging performance in vitro and in vivo. Therefore, it is envisioned that the use of POSS as a multifunctional coating agent can be expanded to other nanoplatforms for multimodal bioimaging and therapeutic applications.

## 7. Conclusions and Perspective

In this review, we highlighted recent research progress on the utilization of polyhedral oligomeric silsesquioxane for biomedical application with an emphasis on drug delivery, photodynamic therapy and bioimaging. In addition, the different strategies for the functionalization of POSS; in particular, the use of click chemistry was summarized. The use of POSS as delivery platform is a burgeoning area of research, which was demonstrated in this review with illustrative examples using anticancer drugs, genes, photosensitizers and contrast imaging agents. Low toxicity, biocompatibility, stability and well-established functionalization techniques are critical features that make POSS attractive in this field. In addition, there are key benefits for the use of POSS in photodynamic therapy: the rigid cage structure acts as a spacer molecule, preventing the aggregation-induced quenching effect which is typically associated with the poor performance of photosensitizers, and POSS can be used as a template to control photosensitizer placement after the nanoparticles have been produced. Finally, the use of POSS as a coating agent for nanoprobes not only renders functionalization capabilities to the material, but can also be used as an effective passivating agent reducing surface trap states that can quench photoluminescence.

While the current results are encouraging and show great potential for future applications of POSS in biomedicine, key breakthroughs are still needed to move this platform forward to clinical applications. The regioselective functionalization of POSS is still a challenge. Novel chemical approaches with controlled and efficient synthesis of POSS that render precise placement of therapeutic/contrast agent and/or functional groups in the molecule are worth developing. Contrary to other silica-based materials such as mesoporous silica or polysilsesquioxane nanoparticles that tend to accumulate in organs like the liver or spleen, [86,87,92], it is expected that POSS will be quickly excreted through the renal excretion pathway due to their size. Nevertheless, there is not a comprehensive and systematic study of the pharmacodynamics and pharmacokinetics of POSS. Preclinical data is critical to push the use of POSS forward to clinic.

We envision that basic understanding and applications of POSS in the area of biomedicine will continue to grow offering future breakthroughs on the synthesis of new POSS platforms, and the clinical implementation of this technology will be developed.

## Figures and Tables

**Table 1:** Different synthetic approaches have been used to modify POSS for biomedical applications.

Ligand(s) for Bioconjugation	Reactive Substituent in POSS	Chemistry Used for Functionalization	Year	Ref.
Carbon nanotubes/paclitaxel/anitbody	Propyl ammonium chloride	Amidation	2015	[41]
Carbon dots	Aminopropyl	Amidation	2015	[42]
BODIPY	Propyl ammonium chloride	Amidation	2005	[43]
Fluorescein	Azidopropyl	Amidation/CuAAC	2010	[33]
Gluconolactone/dansyl chloride	Aminopropyl	Sulfonamide bond	2010	[44]
Tetraphenyl porphyrin	Aminoproppyl	Amidation, urea bond	2020	[27]
Ce6/PEG	Aminopropyl	Amidation	2017	[45]
Fluorescein labeled dipeptide	Propyl ammonium chloride	Amidation	2013	[46]
DOX	Succinic anhydride	Amidation	2016	[47]
DOX/PEG	Succinic anhydride	Amidation	2019	[48]
Porphyrin	Vinyl and hydroxyl	Amidation and CuAAC	2018	[49]
Porphyrin/OPVEs	Vinyl and hydroxyl	Amidation and CuAAC followed by Heck coupling	2020	[50]
FITC/carboxylic acid	Aminopropyl and isobutyl	Amidation	2012	[31]
Perfluorinated alkyl/silica nanoparticles or fluorophore	Propyl ammonium chloride	Amidation	2008, 2009, 2012, 2011	[51,52,53,54]
Peptide/Ce6	Malemic acid	Amidation	2020	[55]
Protoporphyrin IX/linolenic acid	Propyl ammonium chloride	Amidation	2016	[56]
Quantum dots	Propyl ammonium chloride	Amidation	2014	[57]
Quantum dots	Mercaptoropyl	Capping agent	2013	[58]
Ferrocene	Hydrido or hydridodimethylsiloxy	Karstedt’s catalyzed reaction	2012	[59]
Quaternized PDMA—poly (sulfobetaíne)	Propyl ammonium chloride	ARTP	2016	[60]
Poly (fluorinated acrylate -PEG)	Vinyl	RAFT polymerization	2016	[61]
Conjugated oligoelectrolyte	Vinyl	Heck coupling reaction	2010, 2010, 2011, 2011	[62,63,64,65]
Galactose or maltose	Aminopropyl	Amidation	1999	[66]
Glycosides	Vinyl	Thiol-ene click reaction	2004	[67]
Various carbohydrates	Azidopropyl	CuAAC	2010	[68]
Sugar alkynes	Azidopropyl	CuAAc	2010, 2012	[18,69]
Glycosides/Cysteine derivatives	Vinyl	Thiol-ene click reaction	2012	[70]
Poly (lactic acid)	Hydroxypropyl	Microwave assisted ROMP	2018	[71]
BODIPY	Azidopropyl	CuAAC	2010	[18]
PVF	Vinyl	Heck couple reaction	2011	[72]
Dipeptides or tripeptides	Aminopropyl/Hydroxy	Amidation	1998	[73]
Lysine dendritic polypetide	Propyl ammonium chloride	Solution phase peptide synthesis	2007	[74]
Lysine dendritic polypeptide + peptide + DOX	Propyl ammonium chloride	Solution phase peptide synthesis	2009	[75]
Lysine dendritic polypeptide + macrocylic Mn(II) chelates	Propyl ammonium chloride	Solution phase peptide synthesis	2011	[76]
Glutamic acid dendritic polypeptide + biotin + DOX	Propyl ammonium chloride	Solution phase peptide synthesis	2010	[77]
Polypeptide	Azide	CuAAc	2010	[33]
Peptide	Aminoxy	Oxime ligation	2012	[78]
Lysine dendritic polypeptide	Azide	CuAAc	2013	[79]
poly(L-lactide)—poly(ethylene oxide)	3-Hydroxy-3-methylbutyldimethylsiloxy	ROP for the poly-lactide, esterification for poly(ethylene oxide)	2015	[80]
Gd^3+^-DOTA complex	Propyl ammonium chloride	Amidation	2010	[81]
Gd^3+^-DOTA complex	Vinyl	Thiol-ene click reaction	2016	[82]
PEG-cyclodextrin complex	Dimethylsiloxy	Hydrosilylation	2004	[83]

## Sample Availability

Samples of the compounds are not available from the authors.
